# 4261 Insulin Sensitizing Effects of Vitamin D Mediated through Reduced Adipose Tissue Inflammation and Fibrosis: Evidence from a Human Randomized Trial and Mice Studies

**DOI:** 10.1017/cts.2020.304

**Published:** 2020-07-29

**Authors:** Eric Lontchi Yimagou, Sona Kang, Kehao Zhang, Akankasha Goyal, Jee Young You, Evan Rosen, Preeti Kishore, Meredith Hawkins

**Affiliations:** 1 Albert Einstein College of Medicine; 2 University of California, Berkeley; 3 Harvard Medical School

## Abstract

OBJECTIVES/GOALS: Vitamin D [25(OH)D], known to have anti-inflammatory and anti-fibrotic effects in other tissues, may also impact adipose tissue. We designed parallel studies in humans and rodents to define the effects of vitamin D on adipose tissue inflammation and fibrosis, and on systemic insulin resistance. METHODS/STUDY POPULATION: We performed a randomized, double-blinded placebo-controlled trial to examine the effects of repleting vitamin D at to two levels (to >30 ng/ml and to > 50 ng/ml) in 25(OH)D-deficient (<20 ng/ml), insulin resistant, overweight-to-obese humans (n = 19). A comprehensive study of whole-body insulin action was undertaken with euglycemic stepped hyperinsulinemic clamp studies, both before (1st visit) and after administration of vitamin D or placebo (2nd visit and 3rd visit). Adipose tissue fibrosis and inflammation were quantified by ‘real-time’ rt-PCR and immunofluorescence. To determine whether vitamin D’s effects are mediated through adipocytes, we performed hyperinsulinemic clamp studies and adipose tissue analysis in an adipocyte-specific vitamin D receptor knockout (VDR KO) mouse model. RESULTS/ANTICIPATED RESULTS: 25(OH)D repletion (to >30 ng/ml) was associated with reductions in adipose tissue expression of inflammatory (0.6-0.7-fold decreased expression of TNF-α, IL-6, iNOS and PAI-1) and pro-fibrotic (0.4-0.8-fold decreased expression of TGF-β1, HiF1α, Collagen I, V, VI and MMP7) factors, decreased collagen VI immunofluorescence (p = 0.02) and improved hepatic insulin sensitivity in humans, with suppression of endogenous glucose production (EGP) (1.28 ± 0.20 vs 0.88 ± 0.18 mg/kg/min, p = 0.03). Compared to wild type (WT), VDR KO mice exhibited increased adipose tissue expression of several pro-inflammatory (Tnf-α, iNos, Pai-1, Mcp-1 and F4/80; 4-10 fold) and pro-fibrotic genes (Tgf-β1, Collagen VI, and Tsp1; 2-4 fold), in concert with hepatic insulin resistance (EGP 10 ± 3 vs 3 ± 2 mg/kg/min in WT, p = 0.021). DISCUSSION/SIGNIFICANCE OF IMPACT: Collectively, these complementary human and rodent studies establish a beneficial role of vitamin D to improve hepatic insulin resistance, likely by restraining adipose tissue inflammation and fibrosis. Thus, normalizing 25(OH)D levels could have metabolic benefits in targeted individuals. CONFLICT OF INTEREST DESCRIPTION: **N/A**

